# Development of an Alternative Modified Live Influenza B Virus Vaccine

**DOI:** 10.1128/JVI.00056-17

**Published:** 2017-05-26

**Authors:** Jefferson J. S. Santos, Courtney Finch, Troy Sutton, Adebimpe Obadan, Isabel Aguirre, Zhimin Wan, Diego Lopez, Ginger Geiger, Ana Silvia Gonzalez-Reiche, Lucas Ferreri, Daniel R. Perez

**Affiliations:** a Department of Population Health, Poultry Diagnostic and Research Center, University of Georgia, Athens, Georgia, USA; b Virginia-Maryland Regional College of Veterinary Medicine, University of Maryland, College Park, Maryland, USA; Wake Forest University

**Keywords:** T cell immunity, antigenic variation, humoral immunity, influenza B, influenza vaccines, live vector vaccines, orthomyxovirus, viral immunity

## Abstract

Influenza B virus (IBV) is considered a major human pathogen, responsible for seasonal epidemics of acute respiratory illness. Two antigenically distinct IBV hemagglutinin (HA) lineages cocirculate worldwide with little cross-reactivity. Live attenuated influenza virus (LAIV) vaccines have been shown to provide better cross-protective immune responses than inactivated vaccines by eliciting local mucosal immunity and systemic B cell- and T cell-mediated memory responses. We have shown previously that incorporation of temperature-sensitive (*ts*) mutations into the PB1 and PB2 subunits along with a modified HA epitope tag in the C terminus of PB1 resulted in influenza A viruses (IAV) that are safe and effective as modified live attenuated (*att*) virus vaccines (IAV *att*). We explored whether analogous mutations in the IBV polymerase subunits would result in a stable virus with an *att* phenotype. The PB1 subunit of the influenza B/Brisbane/60/2008 strain was used to incorporate *ts* mutations and a C-terminal HA tag. Such modifications resulted in a B/Bris *att* strain with *ts* characteristics *in vitro* and an *att* phenotype *in vivo*. Vaccination studies in mice showed that a single dose of the B/Bris *att* candidate stimulated sterilizing immunity against lethal homologous challenge and complete protection against heterologous challenge. These studies show the potential of an alternative LAIV platform for the development of IBV vaccines.

**IMPORTANCE** A number of issues with regard to the effectiveness of the LAIV vaccine licensed in the United States (FluMist) have arisen over the past three seasons (2013–2014, 2014–2015, and 2015–2016). While the reasons for the limited robustness of the vaccine-elicited immune response remain controversial, this problem highlights the critical importance of continued investment in LAIV development and creates an opportunity to improve current strategies so as to develop more efficacious vaccines. Our laboratory has developed an alternative strategy, the incorporation of 2 amino acid mutations and a modified HA tag at the C terminus of PB1, which is sufficient to attenuate the IBV. As a LAIV, this novel vaccine provides complete protection against IBV strains. The availability of attenuated IAV and IBV backbones based on contemporary strains offers alternative platforms for the development of LAIVs that may overcome current limitations.

## INTRODUCTION

Influenza B virus (IBV) is an enveloped virus with a negative-sense, segmented, single-stranded RNA genome in the Orthomyxoviridae family (1). Eight viral RNA (vRNA) segments are present in the IBV genome, encoding at least 11 proteins (2–4). IBV is considered a major respiratory pathogen of humans, with a well-documented history of epidemics. Although IBV infects all age groups, it causes substantially higher disease burdens in the very young and the elderly (5–8). In the United States, in each season between 2004 and 2011 (excluding the 2009 pandemic), 22 to 44% of all pediatric influenza-related deaths were associated with IBV infections (9). For the United States and Europe, epidemiological evidence in recent years reveals that the burden of IBV is potentially increasing (9, 10). Phylogenetic studies have shown the emergence of two distinct IBV lineages (11–13) that diverged in the 1970s, whereas serological evidence in the 1980s revealed that these lineages had become antigenically distinct. These two lineages, known as the Yamagata (B/Yam) and Victoria (B/Vic) lineages, have virtually no serum cross-reactivity, as evaluated by hemagglutination inhibition (HI) assays (12–14). Although the mutation rate is lower than that observed with influenza A virus (IAV), both IBV lineages continue to undergo antigenic drift as a result of the error-prone characteristics of the viral polymerase and host-mediated antibody pressure.

Vaccines against seasonal influenza viruses are manufactured to confer protection against IAVs and IBVs. For protection, current vaccines rely primarily on the antibody response to the viral surface protein hemagglutinin (HA). The antigenic drift of HA requires influenza vaccines to be updated regularly so that they antigenically match the currently circulating strains (15, 16). Seasonal influenza vaccines traditionally had three influenza virus strains, including two IAV strains (A/H1N1 and A/H3N2) and one IBV strain, representing either the B/Yam or the B/Vic lineage (15). In recent years, however, the two IBV lineages have shown not only seasonal variations but also significant differences in prevalence within different countries, making it extremely difficult to predict which IBV lineage would be predominant in a particular region during a season. Thus, significant antigenic mismatches between seasonal vaccines and circulating IBV strains have been reported in various parts of the world (11–13, 17). In response to this effect, quadrivalent vaccines that incorporate both IBV antigenic lineages in addition to the two IAV strains have been approved and are available (18, 19).

Licensed seasonal influenza vaccines are available in the United States as inactivated influenza vaccines (IIV), recombinant influenza vaccines (rIV), or live attenuated influenza virus (LAIV) vaccines. LAIV vaccines are produced using master donor viruses (MDVs), which carry a series of mutations that restrict virus replication to the upper respiratory tract (with absent or reduced lower respiratory tract replication and minimal clinical signs) (20). In the United States, MDVs were produced by Maassab and colleagues for IBV and IAV by serial passage of the B/Ann Arbor/1/66 (MDV-B) and A/Ann Arbor/6/60 (H2N2) (MDV-A) viruses, respectively, at progressively lower temperatures, resulting in cold-adapted (*ca*), temperature sensitive (*ts*), *in vivo* attenuated (*att*) viruses that grew well at 25°C (21–23). Although both the MDV-B and MDV-A strains show many mutations relative to their respective parental viruses, those that impart the *ca*, *ts*, and *att* phenotypes are located primarily in the polymerase complex (PB1, PB2, PA, and NP). The *ca*, *ts*, and *att* mutations in MDV-B were mapped to PB2 (S630R), PA (V341M), NP (V114A, P410H, and A509T), and M1 (H159Q and M183V), whereas those in MDV-A lie within PB2 (N265S), PB1 (K391E, D581G, and A661T), and NP (D34G) (21–29). These strains have been commercially available under the trade name FluMist in the United States since approval by the Food and Drug Administration (FDA) in 2003. However, a number of issues with regard to the effectiveness of the FluMist vaccine have arisen over the past three seasons (2013–2014, 2014–2015, and 2015–2016), highlighting the critical importance of continued investment in LAIV development in order to develop more-efficacious vaccines.

We have shown previously that in the background of IAVs of either avian or swine origin, incorporation of the single PB2 mutation (N265S) and the three PB1 mutations (K391E, D581G, and A661T) from the MDV-A strain (most of which naturally carry the NP D34G mutation) results in viruses with a *ts* phenotype *in vitro*. However, an adequate *att* phenotype *in vivo* was achieved only after the incorporation into PB1 of a C-terminal epitope tag consisting of a 9-amino-acid sequence derived from the H3 HA gene segment (HA tag) in the context of the *ts* mutations (30–32). Further studies showed that the IAV *att* viruses modified as described above (*ts* mutations plus HA tag) are safe and efficacious as LAIVs for mice, chickens, and swine and are amenable to intranasal (i.n.) administration. For chickens, the IAV *att* vaccines are also suitable for immunization *in ovo* (30–34).

Despite some conserved features in virus structure, genome organization, and the regulation of virus replication and transcription (1, 35–37), IBV and IAV also exhibit a number of important distinctive characteristics, particularly with regard to host range, virus prevalence, and evolutionary dynamics (9, 10, 14). In this report, we explored whether the *ts* mutations plus the HA tag in the IAV *att* strains would result in an *att* phenotype in the context of a prototypic IBV strain. Mutations analogous to those found in our IAV *att* alternative live virus vaccine were introduced into a prototypic B/Vic lineage strain, B/Brisbane/60/2008 (B/Bris). Specifically, mutations were engineered in PB2 (K267S) and PB1 (K391E, E580G, and S660A). In addition, the PB1 segment was modified with the C-terminal HA tag in the presence or absence of temperature-sensitive mutations. Attempts to maintain the PB2 K267S and PB1 K391E mutations stably in B/Bris failed. However, stability studies showed that the E580G and S660A mutations and the C-terminal HA tag were stably maintained over 15 passages in specific-pathogen-free (SPF) eggs and 20 passages in tissue culture cells. Safety and vaccination studies showed that the B/Bris strain with the PB1 segment carrying the E580G and S660A mutations and HA tag (referred to here as B/Bris *att*) was stable, attenuated *in vivo*, and immunogenic. In mouse studies, intranasal immunization with B/Bris *att* resulted in sterilizing immunity against homologous challenge (with B/Bris), and complete protection was achieved following heterologous challenge with a B/Yam lineage strain, B/Wisconsin/01/2010 (B/Wis). These studies show the potential of the alternative LAIV platform for the development of IBV vaccines.

## RESULTS

### Incorporation and maintenance of attenuation markers in an influenza B virus.

The amino acid substitutions associated with the *ts* phenotype of the MDV-A strain have been mapped to the viral RNP (vRNP) complex and have been shown to be transferable to other IAV backbones (38, 39). Early work carried out by our laboratory demonstrated that these substitutions alone do not confer adequate levels of attenuation *in vivo* (defined as either no replication in the lower respiratory tract or a significant reduction in replication). The additional in-frame incorporation of a C-terminal tag in the PB1 segment of IAVs (of either avian or swine origin) was required in order to achieve adequate attenuation *in vivo* (30–32).

**FIG 1:**
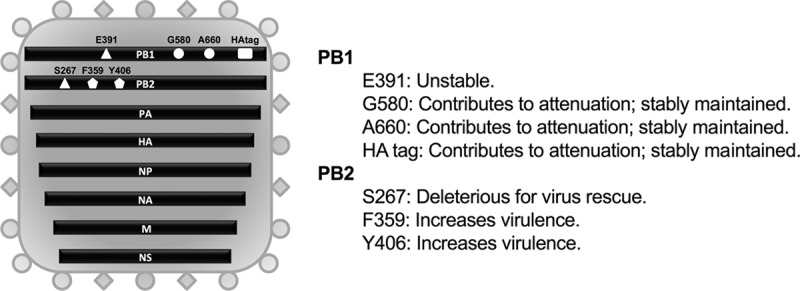
Schematic representation of the IBV genome organization depicting all the modifications tested for IBV attenuation and their respective outcomes. Site-directed mutagenesis and inverse PCR were used to incorporate the attenuation modifications into the PB1 (E391, G580, A660, and HA tag) and PB2 (S267, F359, and Y406) gene segments. The PB1 E391 modification was unstable. PB2 S267 was deleterious for virus rescue, while the PB2 F359 and Y406 substitutions increased IBV virulence. The G580, A660, and HA tag modifications in the PB1 segment were stable and sufficient to confer attenuation (PB1 *att*).

**FIG 2:**
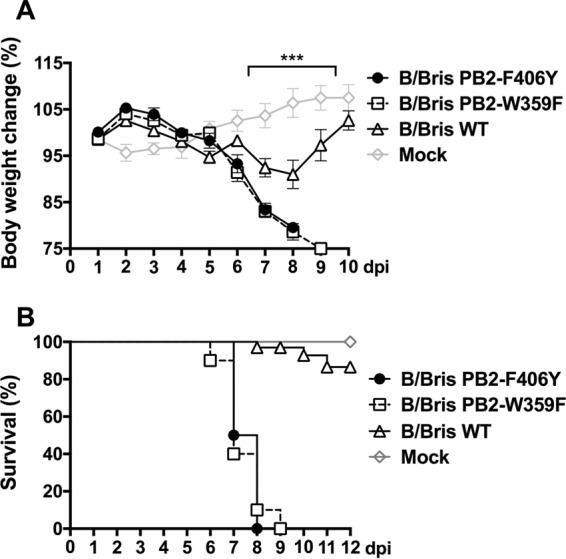
Mutations in PB2 increase IBV virulence. The PB2 F406Y and W359F mutations were individually introduced into the PB2 segment of the B/Brisbane/60/2008 strain by site-directed mutagenesis and were used for virus rescue using reverse genetics. Six-week-old female DBA/2J mice were inoculated i.n. with 10^5^ EID_50_ of the WT RG-B/Bris, B/Bris PB2-F406Y, or B/Bris PB2-W359F virus. The mock-inoculated group received PBS and served as a negative control. The percentage of change in body weight (A) and the survival rate (B) following i.n. inoculation were monitored daily. Plotted data represent means ± standard errors. Two-way ANOVA was performed to calculate *P* values. ***, *P* < 0.001.

**FIG 3:**
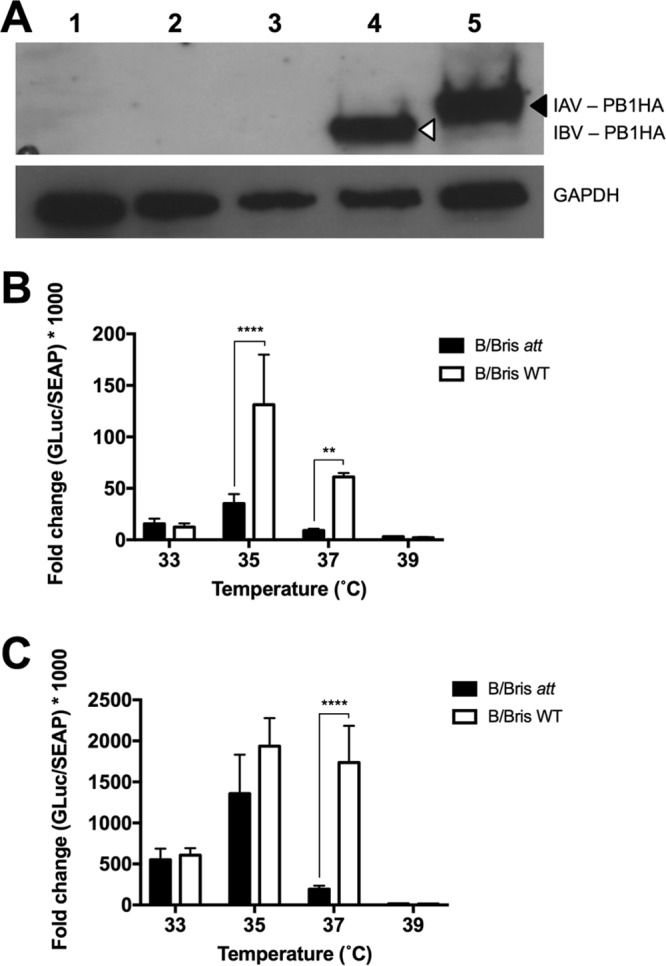
Characterization of the IBV PB1 modifications *in vitro*. (A) HA tag expression in the IAV and IBV carrying a chimeric PB1 HA protein. MDCK cells were either mock inoculated (PBS) (lane 1) or inoculated with WT RG-B/Bris (lane 2), B/Bris *ts* (lane 3), B/Bris *att* (lane 4), or the IAV *att* control (7attWF10:1malH7) (lane 5). PB1 HA chimeric proteins with a molecular mass of >80 kDa are shown: IAV PB1 HA is indicated by a black arrowhead (predicted molecular mass, 88.66 kDa), and IBV PB1 HA is indicated by an open arrowhead (predicted molecular mass, 85.35 kDa). The host cellular protein glyceraldehyde-3-phosphate dehydrogenase (GAPDH; 38.5 kDa) is shown as a gel loading control. (B and C) Virus polymerase activity at different temperatures. 293T cells were transfected with plasmids encoding the PB1 (or PB1 *att*), PB2, PA, and NP and an IBV vRNA-driven luciferase reporter replicon. In addition, a plasmid encoding the secreted alkaline phosphatase (SEAP) under the control of the cytomegalovirus promoter was cotransfected to normalize variations in transfection efficiency. Relative polymerase activity was calculated as the ratio of luciferase activity to alkaline phosphatase activity at 24 hpt (B) or 48 hpt (C). Plotted data represent means ± standard errors. Two-way ANOVA was performed to calculate *P* values. **, *P* < 0.01; ****, *P* < 0.0001.

In this study, we sought to evaluate whether analogous modifications and the resulting *att* phenotype could be transferred to an IBV (Fig. 1). Specifically, using the B/Brisbane/60/2008 strain, we evaluated mutations in PB2 (K267S), PB1 (K391E, E580G, and S660A), and a chimeric PB1 carrying a C-terminal HA tag (in the presence or absence of *ts* mutations). Attempts to rescue an IBV carrying the PB2 K267S mutation resulted in instability at this position; therefore, our efforts were redirected to producing a virus with alternative PB2 mutations. IBVs carrying either the F406Y or the W359F mutation in PB2 were produced, since either one of those mutations affects the activities of the cap-binding protein and the viral polymerase (40). Surprisingly, the B/Bris PB2-F406Y and B/Bris PB2-W359F viruses showed increased virulence in DBA/2J mice (Fig. 2). Therefore, further modifications of the B/Bris genome were focused on the PB1 segment. B/Bris mutant viruses carrying the K391E, E580G, and S660A mutations were produced with or without the C-terminal HA tag (Fig. 3A). Independent virus rescue attempts revealed that the K391E mutation was readily detected in an early virus passage in Madin-Darby canine kidney (MDCK) cells (P2) but was quickly lost after passage in SPF eggs (E2). While the temperature-sensitive phenotype of IAV backbones is determined primarily by the PB1 K391E and E581G mutations (32, 38), the data presented below suggest that the K391E mutation is not critical for attenuation in the context of an IBV. Since the B/Bris mutant virus with the combined E580G, S660A, and HA tag modifications (referred to here as B/Bris *att*) was stable over several passages in either tissue culture cells or SPF eggs, it was further characterized in order to establish whether it showed a *ts* phenotype *in vitro* and whether it was attenuated *in vivo*.

### B/Bris *att* displays a temperature-sensitive phenotype.

*In vitro* characterization of the B/Bris *att* vaccine candidate began by evaluating polymerase activity (measured by levels of Gaussia luciferase [GLuc] expression) in vRNP minigenome assays (Fig. 3B and C). The corresponding components of the IBV vRNP complex, including PB2, PB1 (or its mutant), PA, and NP, and the pBNPGLuc reporter plasmid, along with the pCMV-SEAP plasmid, were cotransfected into 293T cells. GLuc reporter activity was normalized by secreted alkaline phosphatase (SEAP) enzyme activity and served as a surrogate of virus polymerase complex activity. Following transfection, cells were maintained at 33, 35, 37, or 39°C. At 24 and 48 h posttransfection (hpt), supernatants from transfected cells were harvested for the measurement of reporter activity. The results showed that at 33°C, B/Bris *att* and wild-type (WT) RG-B/Bris vRNP complexes displayed similar levels of GLuc reporter activity at either 24 hpt (*P*, nonsignificant [NS]) or 48 hpt (*P*, NS) (Fig. 3B and C, respectively). At 35°C, both vRNP complexes reached peak polymerase activity, although the B/Bris *att* polymerase complex exhibited lower luciferase activity than the WT RG-B/Bris complex (*P*, <0.0001 at 24 hpt). A temperature increase to 37°C further accentuated the statistically significant differences (*P*, <0.001 at 24 h and <0.0001 at 48 h) in levels of luciferase activity between the B/Bris *att* and WT RG-B/Bris polymerase complexes at both time points analyzed, suggesting that modifications in PB1 confer a *ts* phenotype on B/Bris *att*. As expected, at 39°C, both the B/Bris *att* and WT RG-B/Bris polymerase complexes displayed only residual levels of luciferase activity.

**FIG 4:**
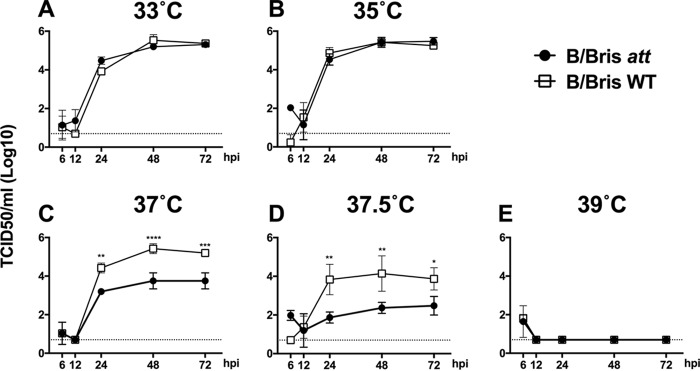
The B/Bris *att* virus displays a *ts* phenotype in growth kinetics *in vitro*. Confluent monolayers of MDCK cells were inoculated at an MOI of 0.01 with either the WT RG-B/Bris or the B/Bris *att* virus and were incubated at 33°C (A), 35°C (B), 37°C (C), 37.5°C (D), or 39°C (E). At 6, 12, 24, 48, and 72 hpi, tissue culture supernatants from inoculated cells were collected for the quantification of virus titers by TCID_50_ assays using the Reed-Muench method. Plotted data represent means ± standard errors. Two-way ANOVA was performed to calculate *P* values. *, *P* < 0.05; **, *P* < 0.01; ***, *P* < 0.001; ****, *P* < 0.0001.

In order to further characterize the impact of PB1 modifications on the *ts* phenotype, the growth kinetics of the B/Bris *att* and WT RG-B/Bris viruses were evaluated. Confluent monolayers of MDCK cells were infected at a multiplicity of infection (MOI) of 0.01, and virus growth kinetics were monitored at 33, 35, 37, 37.5, and 39°C. Overall, the growth kinetics results showed a trend similar to that observed with the vRNP reconstitution assays. The B/Bris *att* and WT RG-B/Bris viruses grew to similar titers at 33°C (Fig. 4A) (*P*, NS, regardless of the time point tested). In contrast to the results of the virus polymerase complex assay, the B/Bris *att* and WT RG-B/Bris viruses also grew to similar titers at 35°C (Fig. 4B) (*P*, NS at 12, 24, 48, and 72 h postinfection [hpi]). At 37°C, the WT RG-B/Bris virus grew to levels similar to those observed at 33°C and 35°C (Fig. 4C). In contrast, the B/Bris *att* virus showed impaired growth at 37°C at 24 (*P* < 0.01), 48 (*P* < 0.0001), and 72 (*P* < 0.001) hpi relative to that of the WT RG-B/Bris virus (Fig. 4C). A half-degree increment in temperature led to a further decrease in the growth of the B/Bris *att* virus, which reached an ∼2-log reduction in the titer at 24 hpi (*P* < 0.01) relative to that of the WT RG-B/Bris virus (Fig. 4D). None of the viruses grew to detectable levels at 39°C, in agreement with the vRNP reconstitution assay data (Fig. 4E). Taken together, these results indicate that modifications introduced into PB1 resulted in a B/Bris *att* virus with a *ts* phenotype *in vitro*.

### The B/Bris *att* virus can be safely administered to mice and is immunogenic.

**FIG 5:**
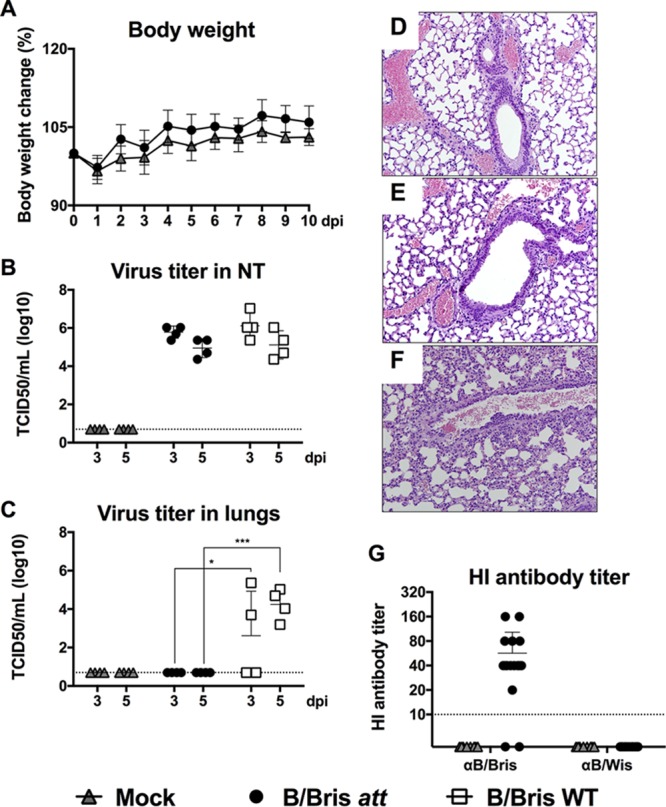
Safety and immunogenicity of the B/Bris *att* virus. Six-week-old female DBA/2J mice were inoculated i.n. with 10^6^ EID_50_ of either the WT RG-B/Bris or the B/Bris *att* virus. The mock-inoculated group received PBS and served as a vaccine negative control. (A) The percentage of change in the body weight of mice was monitored daily following i.n. inoculation with the B/Bris *att* virus. (B and C) Virus replication and tissue tropism of the WT RG-B/Bris and B/Bris *att* viruses in the respiratory tracts of mice. At 3 and 5 dpi, four animals from each group were euthanized, and virus titers in the upper respiratory tracts (nasal turbinates) (B) or lower respiratory tracts (lungs) (C) of the mice were determined by a standard TCID_50_ assay in MDCK cells. Plotted data represent means ± standard errors. Two-way ANOVA was performed to calculate *P* values. *, *P* < 0.05; ***, *P* < 0.001. (D through F) Lungs from mice euthanized 5 days postvaccination were collected and were preserved in 10% formalin for histopathological examination by H&E staining. Images were taken at ×20 magnification. Shown is the H&E staining of lungs from mock-inoculated (PBS) mice (D), B/Bris *att*-vaccinated mice (E), and WT RG-B/Bris-inoculated mice (F). (G) Immunogenicity of the B/Bris *att* virus in mouse sera, measured by an HI assay against the homologous (B/Bris) and heterologous (B/Wis) viruses at 21 dpi.

To investigate whether the *ts* phenotype observed *in vitro* would result in attenuation of the B/Bris *att* virus *in vivo*, we evaluated its safety in mice. Groups of DBA/2J mice were randomly distributed into experimental groups. Each mouse was inoculated i.n. with either phosphate-buffered saline (PBS) (mock inoculation), 10^6^ 50% egg infective doses (EID_50_) of the B/Bris *att* virus, or 10^6^ EID_50_ of the WT RG-B/Bris virus. Clinical signs of disease, body weight changes, and mortality were recorded daily. Body weight monitoring revealed no weight loss following inoculation of the B/Bris *att* vaccine candidate (Fig. 5A). Subsets of mice (*n*, 4/group/time point) were euthanized at 3 and 5 days postinoculation (dpi) in order to determine the levels of virus replication in nasal turbinates (NT) and lungs (the upper and lower respiratory tract, respectively). Both the B/Bris *att* and the WT RG-B/Bris virus replicated well in the upper respiratory tract, as evidenced by virus detection in nasal turbinates (*P*, NS) (Fig. 5B). These results are in agreement with those of vRNP reconstitution assays and growth kinetics experiments performed at 33°C. In contrast, only mice inoculated with the WT RG-B/Bris virus had detectable levels of virus replication in the lower respiratory tract, as shown by significant virus levels in the lungs (Fig. 5C) at 3 (*P* < 0.05) and 5 (*P* = 0.001) dpi. These results are further supported by the histopathology findings for lung samples collected at 5 dpi. Lungs were scored on a scale from 0 to 4, where a score of 4 indicates severe lung pathology. Mice that were either mock inoculated or inoculated with the B/Bris *att* virus had an average whole-lung score of 0 at 5 dpi (Table 1; Fig. 5D and E). In contrast, mice inoculated with the WT RG-B/Bris virus received an average whole-lung score of 2 at 5 dpi, characterized by the presence of mild lesions in the lungs and debris in the bronchial lumen (Table 1; Fig. 5F). These findings are consistent with the vRNP reconstitution assays and growth kinetics experiments, which showed reduced polymerase activity and virus growth at 37°C for B/Bris *att*.

**TABLE 1:** Histopathological findings for mouse lungs

Group	Whole-lung score^*a*^	Major findings
Postinoculation (5 dpi)		
Control	−	
B/Bris *att*	−	Presence of macrophages, minimal alveolitis
RG WT B/Bris	++	Focal lesions, debris in the bronchial lumen, mild alveolitis, and vasculitis
Postchallenge (5 dpc)		
Control + nonchallenged (NC)	−	
Control + B/Bris PB2-F406Y	++	Focal lesions, debris in bronchus and bronchioles, mild necrosis, alveolitis, and vasculitis
B/Bris *att* + B/Bris PB2-F406Y	−	
Control + B/Wis PB2-F406Y	+++	Focal lesions, much debris in bronchus, presence of necrosis and inclusion bodies in bronchioles, severe focal alveolitis, and mild vasculitis
B/Bris *att* + B/Wis PB2-F406Y	+++	Debris in bronchus and bronchioles, moderate focal alveolitis, and minimal vasculitis

a Scores are represented as follows: −, 0; ++, 2; +++, 3.

In order to assess the neutralizing antibody response, all mice were bled at 20 dpi, and HI assays were then performed against the homologous WT B/Bris virus and the heterologous WT B/Wis virus. Nearly all serum samples from B/Bris *att*-inoculated mice (13 of 16) had HI antibody titers of ≥40 against the homologous virus (Fig. 5G), suggesting an adequate and potentially protective antibody response according to current standards for surrogates of protection. Interestingly, 2 of 16 B/Bris *att*-inoculated mice showed no seroconversion by the HI assay. As expected, none of the B/Bris *att*-inoculated mice developed HI antibody titers against the heterologous B/Wis virus (Fig. 5G).

### The B/Bris *att* virus confers sterilizing immunity against homologous challenge (B/Bris PB2-F406Y).

**FIG 6:**
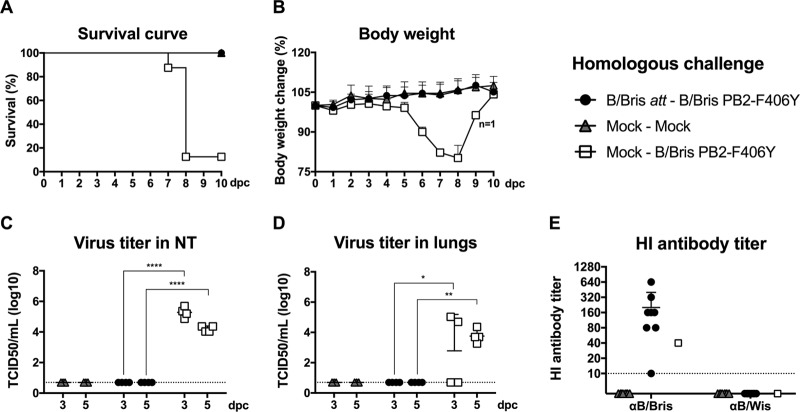
Protective efficacy of the B/Bris *att* virus against challenge with the B/Bris PB2-F406Y virus. Six-week-old female DBA/2J mice were inoculated i.n. with PBS (mock-vaccinated negative control) or with 10^6^ EID_50_ of the B/Bris *att* virus. Three weeks postinoculation, mice were challenged with either PBS (mock) or 10^7^ EID_50_ of the B/Bris PB2-F406Y virus by the i.n. route. (A and B) The survival rate (A) and percentage of change in body weight (B) following challenge with the B/Bris PB2-F406Y virus were monitored daily. (C and D) Virus replication and tissue tropism of the B/Bris PB2-F406Y virus in the respiratory tracts of vaccinated (B/Bris *att*) or negative-control (mock-vaccinated) mice after challenge. At 3 and 5 dpc, four animals from each group were euthanized, and virus titers in the upper respiratory tracts (nasal turbinates) (C) and lower respiratory tracts (lungs) (D) of mice were determined by standard TCID_50_ assays in MDCK cells. (E) The serum antibody response in mice was measured by HI assays against the homologous (B/Bris) and heterologous (B/Wis) viruses at 21 dpc. Plotted data represent means ± standard errors. Two-way ANOVA was performed to calculate *P* values. **, *P* < 0.01; ****, *P* < 0.0001.

To assess the efficacy of the B/Bris *att* vaccine candidate, mock-vaccinated or B/Bris *att*-vaccinated mice (*n*, 16/group) were challenged at 21 dpi with 10^7^ EID_50_ per mouse of the B/Bris PB2-F406Y virus via the i.n. route. The B/Bris PB2-F406Y virus was chosen over the WT B/Bris virus because of its increased virulence (Fig. 2). An additional control group remained unchallenged throughout the study (mock-mock; *n* = 16). B/Bris *att*-vaccinated mice had no apparent signs of disease following the B/Bris PB2-F406Y virus challenge. The changes in body weight in the vaccinated and challenged mouse group were indistinguishable from those seen in the unchallenged control group (Fig. 6B). All B/Bris *att*-vaccinated mice survived challenge with the B/Bris PB2-F406Y virus (*P* < 0.0001) (Fig. 6A). In contrast, control mock-vaccinated mice challenged with the B/Bris PB2-F406Y virus experienced extensive clinical signs of disease and body weight loss. With one exception, all mock-vaccinated mice challenged with the B/Bris PB2-F406Y virus either succumbed to infection or had to be humanely euthanized by day 8 postchallenge (Fig. 6A). Lungs and nasal turbinates were harvested for virus titration from all groups at 3 and 5 days postchallenge (dpc). No virus was detected in the lungs or nasal turbinates of B/Bris *att*-vaccinated mice at either 3 or 5 dpc (Fig. 6C and D). As expected, extensive virus replication in the entire respiratory tract, including both lungs and nasal turbinates, was detected at 3 and 5 dpc in the mock-vaccinated, B/Bris PB2-F406Y-challenged mice (Fig. 6C and D). In addition, B/Bris *att*-vaccinated mice showed an increase in HI antibody titers against the homologous B/Bris virus at 21 dpc (Fig. 6E). Overall, these results suggest that the B/Bris *att* vaccine candidate confers sterilizing immunity against the homologous virus on mice.

### The B/Bris *att* virus protects mice against heterologous challenge (B/Wis PB2-F406Y).

**FIG 7:**
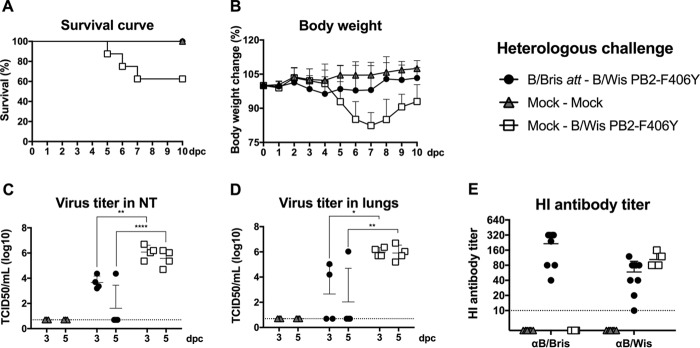
Protective efficacy of the B/Bris *att* virus against challenge with the antigenically heterologous B/Wis PB2-F406Y virus. Six-week-old female DBA/2J mice were inoculated i.n. with PBS (mock-vaccinated negative control) or with 10^6^ EID_50_ of the B/Bris *att* virus. Three weeks postinoculation, mice were challenged either with PBS (mock) or with 10^7^ EID_50_ of the B/Wis PB2-F406Y virus by the i.n. route. (A and B) The survival rate (A) and percentage of change in body weight (B) following challenge with the B/Wis PB2-F406Y virus were monitored daily. (C and D) Virus replication and tissue tropism of the B/Wis PB2-F406Y virus in the respiratory tracts of vaccinated (B/Bris *att*) or negative-control (mock-vaccinated) mice after challenge. At 3 and 5 dpc, four animals from each group were euthanized, and virus titers in the upper respiratory tracts (nasal turbinates) (C) or lower respiratory tracts (lungs) (D) of mice were determined by standard TCID_50_ assays in MDCK cells. (E) Serum antibody responses in mice measured by HI assays against the homologous (B/Bris) and heterologous (B/Wis) viruses at 21 dpc. Plotted data represent means ± standard errors. Two-way ANOVA was performed to calculate *P* values. *, *P* < 0.05; **, *P* < 0.01; ****, *P* < 0.0001.

Live attenuated influenza virus (LAIV) vaccines have been shown to provide more–cross-protective immune responses than inactivated vaccines by eliciting local mucosal immunity in addition to systemic B and T cell-mediated responses. In order to evaluate the extent of cross-protection, mock- or B/Bris *att*-vaccinated mice were challenged at 21 dpi with 10^7^ EID_50_ per mouse of the B/Wis PB2-F406Y virus. All B/Bris *att*-vaccinated mice (*n* = 8) survived the challenge with the heterologous B/Wis PB2-F406Y virus, whereas ∼40% of challenged mock-vaccinated mice (*P* < 0.05) either succumbed to virus infection or were euthanized due to severe clinical disease and/or weight loss (Fig. 7A and B). In contrast, B/Bris *att*-vaccinated mice experienced only mild body weight loss following the heterologous challenge (Fig. 7B). At 3 and 5 dpc, analysis of the lung (*P*, <0.05 at 3 dpc and <0.01 at 5 dpc) and nasal turbinate (*P*, <0.01 at 3 dpc and <0.0001 at 5 dpc) tissues collected showed lower virus loads in B/Bris *att*-vaccinated mice than in challenged mock-vaccinated mice (Fig. 7C and D). As expected, the B/Bris *att*-vaccinated mice experienced a boost in HI antibody titers against the vaccine virus and limited responses to the heterologous B/Wis PB2-F406Y challenge virus as measured at 21 dpc (Fig. 7E). B/Bris *att* immunization did not provide sterilizing immunity following the heterologous B/Wis PB2-F406Y virus challenge. Nonetheless, nearly all B/Bris *att*-vaccinated mice (3 of 4 [75%]) cleared the challenge virus infection by 5 dpc (Fig. 7C and D), suggesting a cross-protective response mediated by nonneutralizing antibodies, T cells, or a combination of both (41).

### B/Bris *att* vaccination reduces lung pathology after IBV challenge.

**FIG 8:**
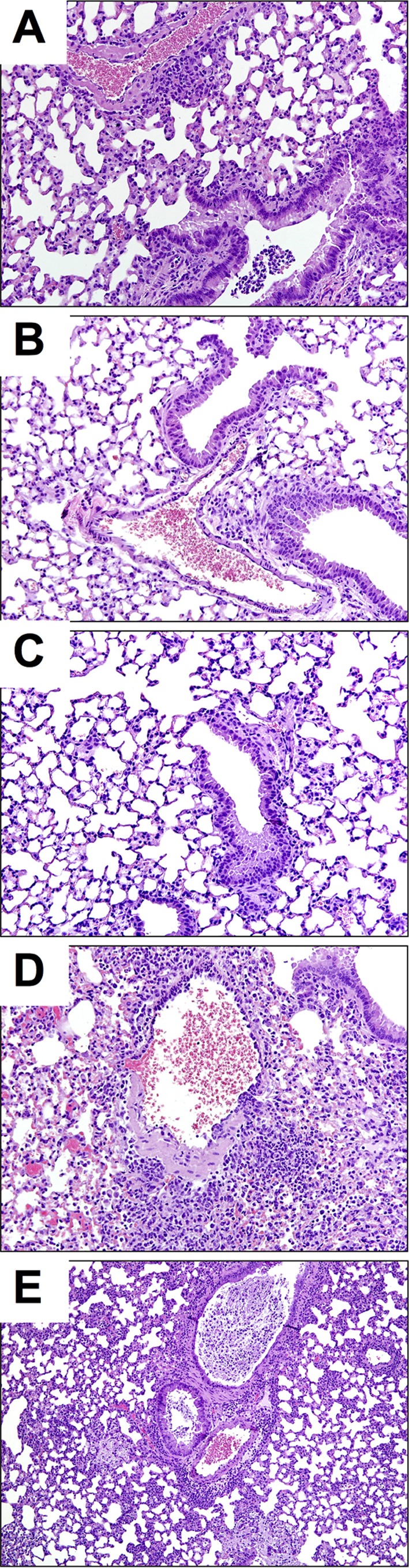
B/Bris *att* vaccination reduces lung pathology after IBV challenge. Lungs from mice euthanized at day 5 postchallenge were collected and were preserved in 10% formalin for H&E staining. Images were taken at ×20 magnification. Shown is H&E staining of lungs from mock-vaccinated mice following challenge with B/Bris PB2-F406Y (A), from B/Bris *att*-vaccinated mice following homologous challenge (B/Bris PB2-F406Y) (B), from mock-vaccinated, mock-challenged (PBS) control mice (C), from mock-vaccinated mice following challenge with B/Wis PB2-F406Y (D), and from B/Bris *att*-vaccinated mice following heterologous challenge (with B/Wis PB2-F406Y) (E).

Histopathological examination of lung samples collected at 5 dpc further confirmed the vaccine-challenge studies. Mock-vaccinated mice challenged with the B/Bris PB2-F406Y virus had a whole-lung score of 2, with the presence of focal lesions, debris in the bronchus and bronchioles, mild necrosis, alveolitis, and vasculitis (Table 1; Fig. 8A). In contrast, lungs from B/Bris *att*-vaccinated mice challenged with the B/Bris PB2-F406Y virus received an overall histopathology score of 0, like lungs from mock-vaccinated, mock-challenged mice (Table 1; Fig. 8B and C). While the B/Wis PB2-F406Y virus was less lethal than the B/Bris PB2-F406Y virus in DBA/2J mice, tissue pathology was more severe. On a scale of lung pathology severity from 0 to 4, mock-vaccinated mice challenged with the B/Wis PB2-F406Y virus had a whole-lung score of 3, with the presence of focal lesions, much debris in the bronchus, necrosis and inclusion bodies in bronchioles, severe focal alveolitis, and mild vasculitis (Table 1; Fig. 8D). Although B/Bris *att*-vaccinated mice challenged with B/Wis PB2-F406Y also received a whole-lung score of 3, the histopathology findings (debris in the bronchus and bronchioles, moderate focal alveolitis, and minimal vasculitis) were milder than those for the challenged mock-vaccinated group (Table 1; Fig. 8E). These results are consistent with the protective role of the B/Bris *att*-stimulated immune response in reducing virus loads and promoting rapid virus clearance. Collectively, these results suggest that the B/Bris *att* vaccine candidate induces a robust protective immune response that can reduce lung pathology in mice following challenge with either antigenically homologous or heterologous IBVs.

### Stability of the B/Bris *att* virus.

Ascertaining genetic stability remains an essential step in the development of LAIV candidates. Thus, the B/Bris *att* virus from the E2 stock was serially passaged at 33°C either in SPF eggs (15 times; serial egg passage 1 [sE1] through sE15) or in MDCK cells (20 times; serial passage 1 [sP1] through sP20). Following serial passage, isolated RNA from both allantoic fluid and the tissue culture supernatant was subjected to whole-IBV-genome amplification and next-generation sequencing (NGS). Sequencing analysis of the PB1 segment confirmed that all modifications introduced (E580G, S660A, and the modified HA tag) were stably maintained following successive passages in SPF eggs and/or MDCK cells (Table 2). In PB1, one mutation at amino acid position 48, E48K (G163A), emerged in sP1 and was maintained through sP20. Interestingly, both sP1 and sP20 viruses contained the PB1 K391E mutation, which was previously lost (or at least below the limit of detection) during preparation of the E2 stock. It is possible that a small virus population containing the PB1 K391E mutation remained in the E2 virus stock. It is tempting to speculate that E391 becomes stabilized in the MDCK cell passage virus with the acquisition of the potentially compensatory PB1 E48K mutation. The sE15 passage virus showed the WT PB1 sequences at both of these positions (E48 and K391); however, it showed one change from the E2 virus stock at position 474, V474I (G1441A) (Table 2). The vRNA extracted from pooled nasal turbinate tissues of B/Bris *att*-vaccinated mice at 3 and 5 dpi revealed the stability of the inserted attenuation markers (E580G, S660A, and the modified HA tag). More importantly, no additional mutations were found within the sequenced PB1 segment from nasal turbinate samples. These results emphasize the safety and stability of the B/Bris *att* virus *in vivo*.

**TABLE 2:** Comparison of amino acid substitutions in IBV *att* strains

IBV protein	Amino acid (nucleotide) substitution(s)^*a*^ in the following virus:										
	B/Bris *att*									Cold-adapted B/Ann Arbor/1/1966^*b*^	Cold-adapted B/USSR/60/1969^*c*^
	Predicted	Amplified in MDCK cells		Amplified in eggs		Serially passaged in MDCK cells		Serially passaged in eggs			
		P1	P2	E1	E2	sP1	sP20	sE1	sE15		
PB2	None	NT	NT	NT	NT	None	None	None	S764 (T2315C)^Syn^	R78Q, M183I, V269I, S630R	3 mutations
PB1	**E391 (G1192), G580 (G1760), A660 (G1999), HA tag**	**E391 (G1192), G580 (G1760), A660 (G1999), HA tag**	**E391 (G1192), G580 (G1760), A660 (G1999), HA tag**	E391K (G1192A)^Rev^, **G580 (G1760), A660 (G1999), HA tag**	E391K (G1192A)^Rev^, **G580 (G1760), A660 (G1999), HA tag**	E48K (G163A)^NonS^, **E391 (G1192), G580 (G1760), A660 (G1999), HA tag**	E48K (G163A)^NonS^, **E391 (G1192), G580 (G1760), A660 (G1999), HA tag**	E391K (G1192A)^Rev^, **G580 (G1760), A660 (G1999), HA tag**	E391K (G1192A)^Rev^, V474I (G1441A)^NonS^, **G580 (G1760), A660 (G1999), HA tag**	R433K, I651V, H751Y	
PA	None	NT	NT	NT	NT	None	E296 (G917A)^Syn^	None	K489Q (A1494C)^NonS^	H160S, S271N, V431M, I495M, Y497H, D589E	2 mutations
HA	None	NT	NT	NT	NT	None	None	None	None	NT	
NP	None	NT	NT	NT	NT	None	K341 (A1083G)^Syn^	None	D377N (G1189A)^NonS^	T55A, I61D, Y129F, V114A, P410H, A509T, I531T, V534I, D535E	
NA	None	NT	NT	NT	NT	None	K371E (A1164G)^NonS^	None	None	NT	
NB	None	NT	NT	NT	NT	None	None	None	None	NT	
BM1	None	NT	NT	NT	NT	None	None	None	None	H159Q, M183V	1 mutation
BM2	None	NT	NT	NT	NT	None	None	None	None	None	1 mutation
NS1	None	NT	NT	NT	NT	None	M106T (T361C)^NonS^	None	None	None	1 mutation
NEP	None	NT	NT	NT	NT	None	None	None	None	None	

a NT, not tested; NonS, nonsynonymous mutation (amino acid change); Syn, synonymous mutation (no amino acid change); Rev, reversion to wild-type amino acid sequence. Reversion mutations are underlined. Modifications introduced to attenuate the virus are in boldface.

b Data from reference 25.

c Data from reference 70 (sequence information not publicly available; only the number of mutations per segment is given).

## DISCUSSION

This study describes the development of an IBV vaccine candidate with a modified PB1 segment, B/Bris *att*. The selection of mutations in PB2 and PB1 and the incorporation of the HA epitope tag into PB1 were based on our previous experience with IAV *att* virus vaccines and the assumption that the analogous amino acids in IBV overlap regions with similar functions. The original goal was to incorporate all possible analogous mutations; however, a limited set of those were tolerated, were stable, and provided the desired attenuated phenotype. The PB2 K267S mutation (N265S in the IAV PB2) was deleterious for virus rescue but not for polymerase activity, which was reduced in the context of PB1 *att* modifications, in minigenome assays (not shown). Surprisingly, alternative mutations in PB2, previously known to impair cap-binding activity (F406Y or W359F), did not attenuate B/Bris virulence in mice but instead increased it over that of the WT strain. It is well established that IBV strains are usually not lethal in mice unless the virus is forcibly adapted in this species. We took advantage of these new findings and used the B/Bris PB2-F406Y virus for lethal challenge in subsequent vaccine efficacy studies. Similarly, the B/Wis PB2-F406Y virus was created for the heterologous challenge, which was more lethal to mice than the WT B/Wis virus but less lethal than the B/Bris PB2-F406Y virus.

Sanger-based sequencing and next-generation sequencing (NGS) were utilized to better understand the stability of the B/Bris *att* virus within and beyond the engineered mutations and HA tag addition. Whole-genome sequences of the early passages of the B/Bris *att* virus obtained from SPF eggs or tissue culture cells were compared to those obtained by passaging the virus multiple times in either SPF eggs or MDCK cells. These sequences were further compared to IBV PB1 sequences obtained from the Influenza Research Database (IRD) (www.fludb.org). More than 4,000 complete IBV PB1 sequences are available in the IRD, but only 793 differ from each other in at least one amino acid, demonstrating remarkable stability for this particular segment.

The PB1 K391E and E580G mutations (the same as in IAV PB1) were tolerated and resulted in adequate polymerase activity; however, the K391E mutation was not always stably maintained in SPF eggs. This mutation was present in viruses grown in MDCK cells (P1) but was lost upon passage in SPF eggs (E2). The reason for the instability of the K391E mutation in SPF eggs is unknown. Further studies will be required, particularly considering the fact that the mutation was stable in virus serially passaged in MDCK cells (sP1 through sP20). The emergence of the PB1 E48K mutation may have played a compensatory role and contributed to maintaining the K391E mutation in MDCK cells. It must be noted that the PB1 E48K mutation also emerged in an alternative virus rescue iteration of B/Bris *ts* (no HA tag) that also maintained the K391E mutation after 9 serial passages in eggs (sE9) (data not shown). The PB1 E48K mutation is unique to the recombinant virus prepared in this study; no other IBV PB1 segment appears to favor such a mutation in nature. How the PB1 E48K mutation could affect the virus phenotype *in vitro* and *in vivo* requires further studies beyond the scope of the present report.

The PB1 E580G mutation was stably maintained in MDCK cells, in SPF eggs, and after replication for at least 5 days in mice. This mutation is not unique to the B/Bris *att* virus, although it does not appear to be highly favored in nature: only 4 of the 793 unique IBV PB1 sequences analyzed contained G580 (∼0.5% if all nonduplicated IBV PB1 sequences available in the IRD are considered). The PB1 G580 mutant strains were not found to be associated with any particular season or location; they represent isolates cocirculating with the more-favored PB1 E580 (wild-type) strains. It would be of interest to determine whether naturally occurring strains containing the PB1 G580 mutation are less fit for growth *in vitro* or *in vivo* than the more-common PB1 E580 strains. If this is the case, it will support the notion that the PB1 G580 mutation contributes to the safety of the B/Bris *att* backbone.

The design of the PB1 S660A mutation (A661T in IAV PB1) was counterintuitive. In IAV, the hydrophobic-to-polar amino acid change in the PB1 A661T mutation contributes to its attenuation. We simply assumed that the opposite change in IBV PB1, the polar-to-hydrophobic amino acid mutation S660A, would be stable and would contribute to the virus's attenuation. In fact, the S660A mutation, unique to the B/Bris *att* virus, was stable over multiple serial passages in either SPF eggs or MDCK cells and contributed to the *ts* phenotype of B/Bris.

The incorporation of the C-terminal HA tag in B/Bris PB1 was also well tolerated and remarkably stable over multiple serial passages in either SPF eggs or MDCK cells. Just as in the modified IAVs, the HA tag does not severely affect the polymerase activity of IBV and is not sufficient to attenuate the virus *in vivo*. The combination of the E580G and S660A mutations with the C-terminal HA tag in PB1 yielded the B/Bris *att* virus with a *ts* phenotype *in vitro* and attenuation *in vivo*. The *ts* phenotype was evident by reduced polymerase complex activity and decreased virus growth kinetics at elevated temperatures (≥37°C). The defective replication of the B/Bris *att* virus in the lungs of mice suggests that the virus is attenuated *in vivo*. The PB1 E580G, S660A, and HA tag modifications were stably maintained not only after multiple passages in either SPF eggs or MDCK cells but also for at least 5 days in viruses isolated from the nasal turbinates of infected mice.

The sE15 B/Bris *att* virus showed one additional amino acid mutation in PB1, V474I. The presence of PB1 I474 is fairly common among IBV field isolates. Neither V474 nor I474 is fixed in the IBV PB1 population. PB1 I474 is present in 100% of strains isolated from 1940 until 2000, including the cold-adapted B/Ann Arbor/1/66 strain. Therefore, it is safe to speculate that the V474I mutation is unlikely to affect the *in vitro* or *in vivo* phenotype of B/Bris *att*. It remains to be determined whether the V474I mutation reflects egg-grown adaptation. In addition, in the sE15 virus, single amino acid mutations were found in PA (K489Q) and NP (D377N). The PA K489Q mutation appears to be unique to the sE15 B/Bris *att* virus, whereas the NP D377N mutation is found in at least one field isolate. The effects of these mutations on virus attenuation are unknown. Other segments remained unchanged; neither amino acid nor nucleotide changes from the predicted wild-type sequence were observed in the sE15 virus. Likewise, no amino acid changes from predicted sequences were observed in the PB2, PA, HA, NP, NB, M, BM, and NEP open reading frames (ORFs) of the sP20 virus. The sP20 virus did show two amino acid changes from the sP1 virus, one in NA (K371E) and one in NS1 (M106T). Neither of these mutations is unique to the sP20 virus, and the NA K371E mutation is fairly common among IBV strains. Next-generation sequencing allows for an unprecedented level of detail in examining the stability of live virus vaccines. Although amino acid changes were observed upon serial passage, the B/Bris *att* virus showed no changes in HA and great stability at the engineered sites in PB1. Other changes do not appear to change the virus's attenuated phenotype.

The growth characteristics of the B/Bris *att* virus are ideal for a safe LAIV vaccine (20, 27, 28, 42). The B/Bris *att* virus was safe in mice, causing no weight loss following inoculation with a high dose of 10^6^ EID_50_. Virus replication was restricted to the upper respiratory tract, as evidenced by virus detection in nasal turbinates but not in lungs. Upon challenge with the homologous B/Bris PB2 F409Y strain, all B/Bris *att*-vaccinated mice showed sterilizing immunity: no challenge virus was detected in either the lungs or the nasal turbinates on any of the days surveyed. Furthermore, no weight loss was seen in any vaccinated mice postchallenge. In contrast, challenged mice in the mock-vaccinated group succumbed to infection, except for one surviving mouse, which nevertheless experienced approximately 20% weight loss postchallenge.

B/Bris *att*-vaccinated mice also survived the challenge with the heterologous B/Wis PB2-F406Y strain. Although the B/Bris *att*-vaccinated mice experienced some mild weight loss following challenge with the heterologous virus, this was in sharp contrast to the challenged mock-vaccinated mouse group, which showed significant body weight loss. The B/Bris *att* protective responses did not confer sterilizing immunity against heterologous challenge. However, most B B/Bris *att*-vaccinated mice generated an immune response that was sufficient to reduce virus replication and promote rapid viral clearance. Local mucosal immunity is likely involved in the mechanism of protection, considering the lack of cross-reactivity in the HI antibody response among antigenically distinct IBV HA lineages. Recently, several studies have demonstrated the development of tissue-resident memory (T_RM_) cells in the lungs after influenza virus infection (43–45) and their critical role in enhanced protection against infection at peripheral entry sites (43, 45, 46). Further studies must be pursued in order to better define the humoral as well as the T cell-dependent responses to the B/Bris *att* vaccine that contribute to cross-protection. It will be particularly important to verify the establishment of T_RM_ cells in mucosal sites following immunization with the B/Bris *att* vaccine and their role in heterologous protection (43, 45).

In the past few years, influenza vaccine development has shifted to finding universal vaccine approaches that will require fewer updates and provide longer-lasting immunity. For the most part, these efforts have overlooked the potential benefits of developing universal vaccines around the concept of a live attenuated virus and have focused largely on eliciting broadly neutralizing antibody responses to highly conserved regions of the HA (47). While recent advances in antigen design to break the immunodominance of HA head and induce broadly protective responses against the HA stalk have been made (47), sequential exposure of the human population to circulating seasonal influenza virus strains may undercut the long term feasibility of these strategies, as a recent study has demonstrated (48). Live attenuated vaccines have been shown to confer better protection than inactivated vaccines due to the stimulation of both humoral (primarily IgG and IgA) and T cell (virus-specific CD4^+^ and CD8^+^ T cell)-mediated immune responses, instead of simply the humoral (IgG) response associated with inactivated vaccines and some universal vaccines (49–51). LAIV vaccines have also been shown to heighten innate immune responses (52–54) and to stimulate cross-protective responses to heterologous or antigenically divergent strains (33, 34). Other advantages include the straightforward administration of LAIV vaccines (needle-free delivery) and the smaller infrastructure capacity required for the manufacture and processing of LAIV vaccines (55).

A number of issues with regard to the effectiveness of the licensed LAIV vaccine in the United States (FluMist) have arisen over the past three seasons (2013–2014, 2014–2015, and 2015–2016). While the reasons for the limited robustness of the vaccine-elicited immune response remain controversial and are a subject of ongoing investigation, possible culprits include the suboptimal performance of the H1N1 component, the inclusion of a second IBV strain, and reduced immunogenicity due to sequential exposure to the same LAIV vaccine backbones over the years (55, 56). These perceived limitations of the currently licensed LAIV vaccine, which is based on the A/Ann Arbor/6/60 (H2N2) and B/Ann Arbor/1/66 cold-adapted (*ca*) backbones, highlight the critical importance of continued investment in LAIV development and create an opportunity to improve the current strategy so as to develop more-efficacious vaccines. A variety of strategies have been employed to develop alternative experimental LAIV vaccines. Such strategies include, but are not limited to, whole and partial gene knockouts, the insertion of foreign sequences, and manipulation of the HA cleavage site. Extensive work has been done on NS, M, NA, and PB2 partial and full knockout vaccines, and while such vaccines have proven effective, there are drawbacks (57–59). For instance, the full knockout vaccines must be grown in non-FDA-approved cell lines that stably express the missing gene in order to achieve the level of growth required of a vaccine strain (59). The strategy of manipulating the HA cleavage site, which has been shown to limit virus growth to the presence of elastase, produces viruses that grow to high titers in approved cell lines but has shown some signs of instability *in vitro* (60, 61). Finally, while some of these strategies have been shown to be effective in the context of IBV (57, 60, 62), most have been tested only in the context of IAV (63).

Our laboratory has developed an alternative IAV strategy, which incorporates the PB2 and PB1 mutations found in the A/Ann Arbor cold-adapted backbone (20, 28). Additionally, our strategy involves an in-frame introduction of a 9-amino-acid HA tag derived from H3 HA at the C terminus of PB1 (30–32). We have demonstrated the safety and efficacy of our strategy *in ovo* as well as in mouse, chicken, and pig models. Furthermore, we have shown that viruses generated using this strategy grow to high titers in established cell lines and SPF eggs (30–32). The current study shows that 2 mutations, rather than the 7 mutations found in the licensed B/Ann Arbor *ca* backbone, in combination with a modified HA tag at the C terminus of PB1, are sufficient to attenuate IBV in the context of a mouse model. Notably, the 2 mutations incorporated into the attenuated IBV strain (E580G and S660A) are analogous to a subset of the mutations present in the A/Ann Arbor *ca* backbone. The availability of contemporary attenuated IAV and IBV (this study) backbones offers an alternative platform for the development of LAIVs that may overcome current limitations.

## MATERIALS AND METHODS

### Cell lines and virus strains.

Madin-Darby canine kidney (MDCK) and human embryonic kidney 293T cells were maintained in Dulbecco's modified Eagle's medium (DMEM) supplemented with 10% fetal bovine serum (FBS) and 1% antibiotic/antimycotic solution (Sigma-Aldrich, St. Louis, MO). Cells were propagated at 37°C in a humidified incubator under a 5% CO_2_ atmosphere. The B/Brisbane/60/2008 (WT B/Bris) and B/Wisconsin/01/2010 (WT B/Wis) influenza B virus strains were a gift from Ruben Donis, Centers for Disease Control and Prevention, Atlanta, GA. Virus stocks were amplified in specific-pathogen-free (SPF) embryonated chicken eggs (B&E Eggs, York Springs, PA, or Charles River, Norwich, CT) and were stored at −80°C. The viruses used in this report are summarized in Table 3.

**TABLE 3:** Influenza viruses used in the present study

Virus	Description	Designated abbreviation
B/Brisbane/60/2008	Wild-type B/Brisbane/60/2008 virus	WT B/Bris
Reverse-genetics B/Brisbane/60/2008	Wild-type B/Brisbane/60/2008 virus generated by reverse genetics	WT RG-B/Bris
B/Wisconsin/01/2010	Wild-type B/Wisconsin/01/2010 virus	WT B/Wis
B/Brisbane/60/2008 *ts*	B/Brisbane/60/2008 virus carrying temperature-sensitive mutations (E580G and S660A) in PB1	B/Bris *ts*
B/Brisbane/60/2008 *att*	B/Brisbane/60/2008 virus carrying temperature-sensitive mutations (E580G and S660A) and an HA tag in PB1	B/Bris *att*
B/Brisbane/60/2008 PB2 W359F	B/Brisbane/60/2008 virus carrying the W359F mutation in PB2	B/Bris PB2-W359F
B/Brisbane/60/2008 PB2 F406Y	B/Brisbane/60/2008 virus carrying the F406Y mutation in PB2	B/Bris PB2-F406Y
2 B/Wisconsin/01/2010:6 B/Bris PB2 F406Y	Reassortant virus containing the HA and NA genes of B/Wisconsin/01/2010 and the internal gene constellation of the B/Brisbane/60/2008 F406Y virus	B/Wis PB2-F406Y
1 A/mallard/Alberta/24/01 (H7N3):7 A/guinea fowl/Hong Kong/WF10/1999 (H9N2)	Reassortant virus containing the HA of A/mallard/Alberta/24/01 (H7N3) and 7 remaining genes of the attenuated A/guinea fowl/Hong Kong/WF10/1999 (H9N2) virus (WF10*att*)	7attWF10:1malH7

### Animal use and compliance.

Five- to 6-week-old female DBA/2J mice (Charles River, Frederick, MD, or The Jackson Laboratory, Bar Harbor, ME) were used in all mouse experiments. All animal studies were performed under animal biosafety level 2 (ABSL-2) containment conditions according to protocols approved by the respective Institutional Animal Use and Care Committees (IACUC) at the University of Georgia and the University of Maryland, College Park. Mice that experienced significant weight loss (21 to 25%) or scored 3 or higher on a 4-point scale of disease severity were humanely euthanized.

### Cloning and site-directed mutagenesis.

The reverse genetics system for the WT B/Bris strain has been described elsewhere (35). The HA and NA surface gene segments of WT B/Wis were cloned into the bidirectional cloning vector pDP2002 by standard cloning techniques as described previously (32, 35). To generate the attenuated PB1 segment (PB1 *att*), a modified HA tag was first cloned in-frame at the C terminus of PB1 by inverse PCR. The cloning strategy was based on the HA tag cloning procedure described previously (32), with minor modifications. Primers were designed such that the original stop codon in PB1 was mutated to an alanine (A), and a new stop codon was introduced following the modified HA tag. Additionally, the codon for the last amino acid present in the wild-type PB1 sequence (isoleucine [I]) was repeated immediately before the introduced alanine (A) codon. Thus, the entire amino acid sequence introduced into PB1 was IAYPYDVPDY, with the final 8 amino acids (underscored) corresponding to the modified HA tag. Subsequently, the K391E, E580G, and S660A mutations were introduced into PB1 via site-directed mutagenesis using PCR. The K267S, F406Y, or W359F mutation was introduced into the PB2 segment of the B/Brisbane/60/2008 genome by site-directed mutagenesis with the QuikChange II XL kit (Agilent, Santa Clara, CA). PCRs were performed with either *Pfu* Ultra DNA polymerase AD (Agilent, Santa Clara, CA) or Phusion High Fidelity DNA polymerase (New England BioLabs, Ipswich, MA). All plasmids constructed were sequenced, and no unwanted mutations were identified. A schematic overview of all the modifications introduced into the IBV genome and their respective outcomes is given in Fig. 1.

### Generation of recombinant viruses by reverse genetics.

Viruses were rescued using a coculture of 293T and MDCK cells as described previously (64). To generate the B/Bris *att*, B/Bris PB2-F406Y, and B/Bris PB2-W359F viruses, the respective mutant plasmid was paired with the 7 remaining WT B/Bris plasmids. The B/Wis PB2-F406Y virus is a 2:6 reassortant carrying the surface genes of WT B/Wis and the internal gene constellation of the B/Bris PB2-F406Y virus. Following transfection, transfected cells were incubated at 35°C. After 24 h of incubation, the medium was replaced with Opti-MEM I (Life Technologies, Carlsbad, CA) containing 1 μg/ml tosylsulfonyl phenylalanyl chloromethyl ketone (TPCK)-trypsin (Worthington Biochemicals, Lakewood, NJ) and antibiotic/antimycotic solution. Following virus rescue (passage 0 [P0]), the tissue culture supernatant was used to inoculate fresh MDCK cells to amplify the virus once (P1). Viruses in P1 were used for further amplification in MDCK cells (P2) and then in SPF eggs (E1). The viruses obtained in E1 were used to grow virus stocks in SPF eggs (E2). P1, P2, E1, and E2 virus amplifications were carried out at 33°C. Virus stocks were titrated both by 50% tissue culture infectious doses (TCID_50_) and by 50% egg infectious doses (EID_50_). Virus titers were determined by the Reed-Muench method (65).

### Stability of attenuated influenza B virus.

All the virus stocks were sequenced by either Sanger sequencing or next-generation sequencing (NGS), or both, to assess the presence of the inserted mutations in rescued viruses. To ascertain the stability of attenuation markers, the B/Bris *att* virus (E2) was serially passaged either as many as 20 times in MDCK cells or as many as 15 times in SPF eggs. RNA from tissue culture supernatants and allantoic fluid, respectively, was purified using the RNeasy minikit (Qiagen, Valencia, CA). Isolated RNA served as the template for the amplification of the whole IBV genome by one-step reverse transcription-PCR (RT-PCR) (66) and NGS using the Illumina MiSeq platform (67). Additionally, pooled nasal turbinate homogenates obtained from mice at 3 and 5 days postvaccination (dpv) were used for RNA isolation. The PB1 segment was amplified using two-step RT-PCR (68) and was sequenced to verify the stability of the B/Bris *att* virus following immunization.

### Minigenome assays.

To assess viral polymerase activity, a Gaussia luciferase (GLuc) reporter plasmid (pBNPGLuc) was constructed. The open reading frame (ORF) of GLuc was amplified by PCR from pGLuc-Basic (New England BioLabs, Ipswich, MA) and was subcloned into pDP2002. The plasmid was engineered such that the GLuc ORF was flanked by the 5′ and 3′ untranslated regions (UTRs) of the IBV NP segment. This construct was further modified by removing the pCMV promoter immediately upstream of the 5′ UTR. The resulting plasmid was verified by sequencing, which revealed no unwanted modifications. The vRNP minigenome assay was reconstituted in 293T cells cotransfected with pCMV-SEAP, pBNPGLuc, and B/Bris PB2, PB1, PA, and NP plasmids using the TransIT-LT1 transfection reagent (Mirus, Madison, WI) according to the manufacturer's instructions. The pCMV-SEAP plasmid expresses the secreted alkaline phosphatase and was used to normalize for transfection efficiency. At 24 and 48 hpt, tissue culture supernatants from transfected cells were collected to measure reporter activity using a Victor X3 multilabel plate reader (PerkinElmer, Waltham, MA). GLuc activity was assessed using the Biolux Gaussia luciferase assay kit (New England BioLabs, Ipswich, MA), while phosphatase activity was measured using the Phospha-Light SEAP reporter gene assay system (Life Technologies, Carlsbad, CA). Experiments were carried out independently at least twice, and all transfection conditions were tested in triplicate per experiment.

### Western blotting.

Confluent MDCK cells were inoculated with influenza viruses (WT RG-B/Bris, B/Bris *ts*, B/Bris a*tt*, or 7attWF10:1malH7) at an MOI of 1 for 1 h at 37°C. The 7attWF10:1malH7 virus is a reassortant IAV *att* control virus that has been described previously (32). Following inoculation, infected cells were incubated at 33°C for 20 h. The supernatant was then removed, and cells were lysed with 150 μl of Laemmli buffer containing β-mercaptoethanol (Bio-Rad, Berkeley, CA). Cell lysates were boiled for 7 min, followed by brief sonication. Proteins were separated on a 4-to-20% sodium dodecyl sulfate-polyacrylamide gel electrophoresis (SDS-PAGE) gel and were transferred to nitrocellulose membranes (Bio-Rad, Berkeley, CA) for immunoblot analysis. The membranes were blocked in 5% molecular-grade nonfat dry milk (NFDM; Bio-Rad, Berkeley, CA) for 2 h at room temperature, followed by incubation with a mouse primary antibody against glyceraldehyde-3-phosphate dehydrogenase (GAPDH) (Santa Cruz Biotech, Dallas, TX) for 2 h at room temperature or a mouse primary antibody against the HA tag (Cell Signaling Technologies, Danvers, MA) overnight at 4°C. After washing, the membranes were incubated with a goat anti-mouse IgG antibody conjugated to horseradish peroxidase (Southern Biotech, Birmingham, AL). Immunoreactive proteins were visualized by autoradiography using an enhanced chemiluminescence reagent (Clarity Western ECL substrate; Bio-Rad, Berkeley, CA).

### *In vitro* growth kinetics.

The growth kinetics of the WT RG-B/Bris and B/Bris *att* viruses were compared at different temperatures. Confluent monolayers of MDCK cells were inoculated at an MOI of 0.01 for each virus. At 6, 12, 24, 48, and 72 hpi, tissue culture supernatants from inoculated cells were collected for virus titer quantification. Virus titers were determined by TCID_50_ assays using the Reed-Muench method (65). Experiments were carried out independently at least twice, and all conditions were tested in triplicate per experiment.

### Safety assessment of B/Bris PB2 mutant viruses.

To evaluate the impact of the PB2 F406Y and W359F mutations on increased IBV virulence, DBA/2J mice (Charles River, Frederick, MD) were randomly distributed into 4 groups (*n*, 10 per group). Each mouse was anesthetized with isoflurane and was subsequently inoculated via the intranasal (i.n.) route. Group 1 received 50 μl of PBS and served as a control group. Each mouse in group 2, 3, or 4 was inoculated with 10^5^ EID_50_ of the B/Bris PB2-F406Y, B/Bris PB2-W359F, or WT RG-B/Bris virus, respectively, in 50 μl of PBS. Clinical signs of disease, body weight changes, and mortality were monitored daily.

### Vaccination and challenge studies.

To evaluate the immune response and protective efficacy of B/Bris *att*, DBA/2J mice (Charles River, Frederick, MD, or The Jackson Laboratory, Bar Harbor, ME) were randomly distributed into 5 groups (*n*, 24 per group). Each mouse was anesthetized with isoflurane and was subsequently inoculated i.n. Groups 1, 2, and 3 received 50 μl of PBS alone as mock-vaccinated control groups. Groups 4 and 5 were inoculated with 10^6^ EID_50_ per mouse of the B/Bris *att* virus in 50 μl of PBS. An additional group (*n* = 8) received 10^6^ EID_50_ per mouse of the WT RG-B/Bris virus and served as a control for *in vivo* evaluation of the attenuated phenotype of B/Bris *att*. At 3 and 5 days postinoculation (dpi), 3 or 4 animals from each group were humanely euthanized for tissue collection. Nasal turbinates and lungs were collected for virus titer quantification and histopathological examination. The day before challenge (20 dpi), mice were bled from the submandibular vein to measure neutralizing antibody responses. At 21 dpi, group 1 remained unchallenged, while groups 2, 3, 4, and 5 (*n*, 16 per group) were challenged i.n. with 10^7^ EID_50_ per mouse of either the B/Bris PB2-F406Y or the B/Wis PB2-F406Y virus in 50 μl of PBS. Natural isolates of IBV are usually not lethal to mice unless the virus is serially adapted in this species or a high virus dose is used for inoculation. The increased virulence observed in mice inoculated with the B/Bris PB2-F406Y or B/Bris PB2-W359F virus (Fig. 2A and B) prompted us to select the B/Bris PB2-F406Y virus for lethal challenge in vaccine efficacy studies. Similarly, the B/Wis PB2-F406Y virus was created for heterologous (antigenically distinct) challenge. At 3 and 5 days postchallenge (dpc), 4 mice per group were humanely euthanized for tissue collection as described above. At 21 dpc, all remaining mice (*n*, 8/group) were bled from the submandibular vein for serology. Clinical signs of disease, body weight changes, and mortality were monitored daily throughout the study.

### HI assay.

Serum samples collected at 20 dpi and at 21 dpc were assayed for the presence of neutralizing antibodies by an HI assay as described elsewhere (69) using the WT B/Bris or WT B/Wis virus as the antigen.

### Histopathology.

At 5 dpc, lungs from mice euthanized postvaccination and postchallenge were collected and preserved in 10% formalin for histopathological examination by hematoxylin-and-eosin (H&E) staining. Tissue sections from bronchi/bronchioles, pulmonary vasculature, and alveoli were examined, and the overall extent of pulmonary lesions was scored on a scale from 0 to 4, based on increasing levels of tissue damage and inflammation. Representative images of lung histopathology were taken at ×20 magnification.

### Statistical analyses.

All data analyses were performed using GraphPad Prism software, version 7 (GraphPad Software Inc., San Diego, CA). All *in vitro* assays were performed at least twice in triplicate. For multiple comparisons, two-way analysis of variance (ANOVA) was performed, followed by a *post hoc* Bonferroni test. Differences in survival curves were analyzed using the log rank test. A *P* value below 0.05 was considered significant.
